# Crescent Moon Image as a Peculiar Complication During Percutaneous Coronary Intervention of an In-Stent Chronic Total Occlusion

**DOI:** 10.5935/abc.20170073

**Published:** 2017-08

**Authors:** Mohsen Mohandes, Jordi Guarinos, Cristina Moreno, Sergio Rojas, Alfredo Bardají

**Affiliations:** Interventional Cardiology Unit - Cardiology Division - Joan XXIII University Hospital - Universitat Rovira Virgili, Tarragona - Spain

**Keywords:** Percutaneous Coronary Intervention, Coronary Occlusion, Drug Eluting Stents, Ultrasonography, Interventional, Cardiac Catheters

## Case

**Figura 1:**
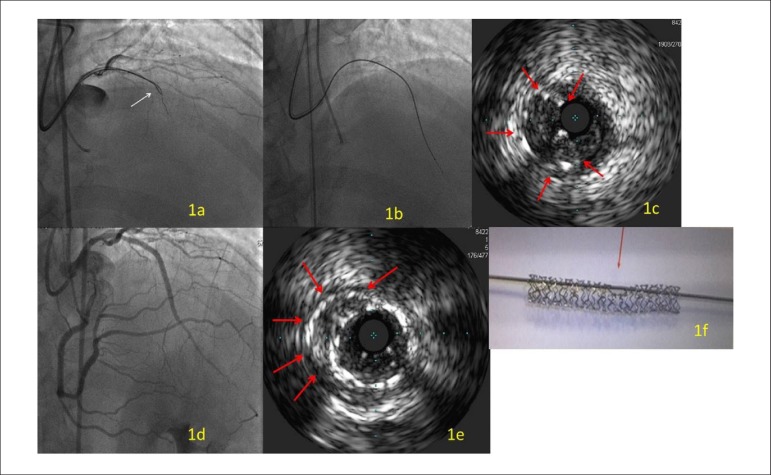
1a) Fio-guia Confianza Pro 9 penetrando o segmento ocluído do stent com a técnica de guia-paralelo. 1b) Micro cateter Tornus passado com sucesso pelo segmento ocluído do cateter e levado até a porção distal da artéria. 1c) Pontas das setas limitam o ponto sub-expandido do stent sem outras distorções após a dilatação do balão. Sonda de IVUS nesse ponto está posicionada no verdadeiro lúmen, mas fora do stent previamente implantado. 1d) Recanalização bem-sucedida da artéria coronária descendente anterior esquerda após implante de diversos stents eluidores de fármacos, embora haja observação de embolização distal. 1e) imagem em lua crescente após implantação de novo stent, esmagando o stent prévio. 1f) Modelo de stent sub-expandido mostra como o fio-guia pode deixar o stent e, após, adentrar no lúmen do stent.

55-year-old male with history of ischemic cardiomyopathy with previous percutaneous coronary intervention (PCI) in the anterior descending coronary artery (LAD) middle segment was admitted to our hospital for chest pains. A new coronary angiogram showed in-stent chronic total occlusion (ISCTO) of LAD receiving collaterals from right coronary artery (RCA). Left circumflex (LCX) was totally occluded and RCA presented a significant mid-segment lesion. Complete percutaneous revascularization was planned. First attempt to recanalize the LAD failed because the lesion obstructed the passage of the balloon, so a second dedicated attempt was planned. Bilateral injection using radial and femoral arteries was used, and a Confianza Pro 9 (Asahi Intecc, Japan) guidewire was progressively advanced through the ISCTO (Figure 1a) and the wire's position in true lumen was verified by contralateral injection. Considering the lesion, which impeded the balloon's passage, a microcatheter Tornus (Asahi Intecc, Japan) was utilized to penetrate and advance through and past the occlusion (Figure 1b). After balloon predilatation, intravascular ultrasound sonography (IVUS) verified the position of the wire in a very short segment into true lumen, but outside the previously implanted stent (Figure 1c). Several drug eluting stents (DES) were implanted and the artery was successfully recanalized, although a very distal embolization was detected (Figure 1d). A new IVUS examination showed partial crush of the previous stent in the shape of a crescent moon (Figure 1e).

This rare complication is likely due to an underexpanded stent point in the first procedure. The guidewire in this point got out through a stent strut but remained within the true lumen (Figure 1f). After balloon predilatation and stent implantation, the former stent was crushed in its underexpanded point. This is a potential complication which could happen during ISCTO-PCI and a careful examination by IVUS before stent implantation can localize the wire bias. There is a difficult but feasible manoeuvre consisting of reintroducing a new wire into the stent lumen guided by IVUS, which can potentially avoid the above complication.

